# Antenatal Care Services in Sudan Before and During the 2023 War: A Review Article

**DOI:** 10.7759/cureus.51005

**Published:** 2023-12-23

**Authors:** Amani Abdelmola

**Affiliations:** 1 Family and Community Medicine, Jazan University, Jazan, SAU

**Keywords:** pregnancy care, prenatal care, sudan khartoum, sudan conflict, maternal mortality, war in sudan, anc during armed conflicts, political instability, antenatal care

## Abstract

Antenatal care (ANC) is provided by skilled healthcare professionals to pregnant women to ensure the best health conditions for both mother and baby during pregnancy. It includes risk identification, prevention, management of pregnancy-related diseases, health education, and health promotion. Antenatal care has a great effect on vital health indicators such as maternal and neonatal mortality by identifying and treating pregnancy-related complications. Political instability and armed conflict have seriously affected the health system, which has catastrophic implications for pregnant women's health. This review aimed to summarize the literature on ANC in Sudan before and during political instability and war by highlighting its effect on maternal mortality, coverage, care providers, quality of care, accessibility, and utilization. Other aspects of this review are the ANC components and service provision during the war. In addition, the author tried to identify the gaps and point out the future research needs in Sudan. A total of 58 articles about ANC in Sudan have been reviewed through PubMed, Google Scholar, ResearchGate databases, and other search tools. The keywords used were "antenatal care", "coverage", "service providers", "service quality", "accessibility and utilization", "components", and "ANC during the war". All the keywords were followed by “Sudan” to confine the search. According to the reviewed data, ANC services in Sudan, even during normal political situations, were not sufficient and of poor quality in most of the reviewed regions. The political instability and armed conflicts worsened the situation, and it became catastrophic. To improve the accessibility and quality of ANC services, we will need the collaboration of all stakeholders to address the health needs of vulnerable groups, people in remote rural areas, and nomadic communities towards providing the required health services in general and ANC in particular. On the other hand, an important aspect of this development is the availability of skilled healthcare providers and the adoption, revision, and updating of working procedure guidelines to match the needs of the local communities. The main shareholders are the local communities; they must be empowered and involved by raising their awareness. Then, effective, punctual, and applicable contingency plans should be ready for any unfortunate crises.

## Introduction and background

International human rights law contains fundamental obligations on states to enable women and adolescent girls to survive pregnancy and childbirth as part of their enjoyment of sexual and reproductive health and rights and to live lives of dignity [1]. Based on this understanding of the obligation to provide the necessary healthcare to women during pregnancy, antenatal care (ANC) is of great importance and must be given special attention. Providing reproductive health services in Sudan has always been a real challenge due to many reasons, including the nonexistence of trained health personnel, insufficient funding necessary to provide the service, and sometimes inadequate women’s awareness of the importance of the service. In conditions of political instability and war, rights in general and health services in particular deteriorate, and one of their victims is a pregnant woman. In this review, we will focus on the ANC in Sudan during peaceful circumstances, during the war conditions currently taking place, and the resulting impact. The review covers different dimensions such as the effect of ANC on maternal mortality, coverage, care providers, quality of care, accessibility and utilization, the components of ANC, and service provision during the war.

Antenatal care is the care provided by skilled healthcare professionals to pregnant women to ensure the best health conditions for both mother and baby during pregnancy. It includes risk identification, prevention, management of pregnancy-related diseases, health education, and health promotion [2]. It is considered an opportunity to communicate with and support women, families, and communities at a critical time in the course of a woman’s life to ensure a positive pregnancy experience through a normal pregnancy and safe delivery for a healthy baby. According to the WHO, a positive pregnancy experience is preserving physical and sociocultural normality, ensuring a healthy pregnancy for mother and baby (including preventing and treating risks, illness, and death), having a safe delivery, and achieving positive motherhood (including maternal self-esteem, competence, and autonomy) [2]. The perfect routine for ANC visits was recommended by the WHO as once a month during the first 28 weeks, twice a month up to 36 weeks, and then weekly until delivery. To expand women’s care experience and diminish perinatal mortality, a minimum of four visits are suggested: the first one at about 16 weeks, the second one between 24 and 28 weeks, the third at 32 weeks, and the fourth at 36 weeks [2]. The essential components of ANC include the promotion of health and well-being via education and support for nutrition, nutritional supplements, immunization, a healthy lifestyle, recognition of danger signs, identification of potential complications and risk factors, screening for health conditions that can affect the mother and fetus, and birth preparedness. According to the WHO, in an ideal world, "every pregnant woman and newborn receives quality care throughout the pregnancy, childbirth, and postnatal period" [3]. The purpose of this review was to summarize the literature on ANC in Sudan prior to and during political instability and war, focusing on the impact on maternal mortality, coverage, care providers, quality of care, accessibility, utilization, ANC components, and service provision during the war. In addition, the author tried to identify the gaps and point out the future research needs in Sudan.

## Review

Antenatal care and maternal mortality

The maternal mortality rate in Sudan has shown a significant decline since 2001, when it was 636 deaths per 100,000 live births, and in 2020, it was estimated to be 295 deaths per 10,000 live births, which is still one of the highest rates globally. About one-quarter of births are not attended by skilled healthcare providers [4].

Worldwide, preventable causes related to pregnancy and childbirth caused the deaths of nearly 800 women daily during 2020; about 95% of all maternal deaths happened in low and lower-middle-income countries [5]. Sub-Saharan Africans suffer from the highest maternal mortality ratio: 533 maternal deaths per 100,000 live births, representing 68% of all maternal deaths per year worldwide [6]. By identifying and treating pregnancy-related complications, ANC directly and indirectly reduces maternal and perinatal morbidity and mortality. Antenatal care helps by identifying women and girls who are more likely to experience complications during delivery and making sure they are referred to the appropriate level of care [7]. On the other hand, child and infant mortality in Sudan is among the highest in the region and the world. According to the study, which analyzed data from the Sudan Household Health Survey 2nd round (SHHS2) in 2010 to investigate factors associated with neonatal and infant mortality and look for interventions for reduction, it was concluded that ANC is one of the most important public health interventions because it adopts a risk-factor-based approach to detect pregnancy complications early and is eligible to deal with them adequately [8]. A systematic review and meta-analysis of prevalence and contributing factors regarding first-trimester ANC visits among African women concluded that few African women initiate first-trimester ANC contact, mostly due to the shortage of maternal healthcare services. Residency, age, educational level, primiparity, having planned pregnancies, and the working status of women were the factors with significant associations. A comprehensive plan is needed to expand maternal health, including all partners and stakeholders in healthcare provision, considering raising women’s educational levels, especially for rural women, and reducing unintended pregnancies, and low socioeconomic status [9]. In Sudan, the effect of ANC on maternal mortality during 2005-2009 in Kassala, eastern Sudan, was assessed by investigating the incidence and causes of maternal deaths. There were 132 maternal deaths and 20,485 (644/100,000) live births. Septicemia, preeclampsia, eclampsia, hemorrhage, anemia, viral hepatitis, and malaria were the most frequent causes of maternal mortalities. Lack of ANC was one of the predictors of maternal death in addition to primipara, illiteracy, and rural residence [10]. Other researchers were assessing the epidemiology of maternal mortality and poor perinatal outcomes in different regions of Sudan. Maternal mortality ranged from 442 (146/33,034) to 640 (63/9,841) per 100,000 births and 9.2% perinatal deaths in the different regions of Sudan. Most of these were due to causes that can be prevented by providing proper, high-quality ANC services with special consideration toward nutrition, malaria prevention, and other communicable diseases [11]. In Wad Madani Teaching Hospital, Sudan, in 2006, the effect of ANC on the probability of neonatal survival at birth was assessed. Results demonstrated that the frequency of stillbirth was very high (31.11%) among women in the categories of "bad health" and "no ANC." This confirms the fact that the adequacy of ANC is strongly and consistently associated with birth outcomes and is the conclusive variable in pregnancy-required outcomes [12]. Many researchers link the rise in maternal and newborn mortality rates in Sudan to the traditional practices of female circumcision and its accompanying complications, in addition to limited access to health services, including pregnancy care, especially in some rural communities and among those displaced from wars, which also leads to high rates of negative outcomes in the period. Perinatal and associated disability in Sudan, as in many countries in sub-Saharan Africa [13], is another aspect of the effect of ANC on maternal mortality in grand multiparity. This was assessed in Khartoum, Sudan, in 2019 to determine the maternal and perinatal outcomes of grand multiparity. Since grand multiparity is prevalent in developing countries, especially in Sub-Saharan Africa, with a high prevalence varying between 17% and 33%, and is considered a major health problem that leads to adverse maternal and perinatal outcomes in other contexts, it has been attributed to factors such as poverty, unavailability of healthcare resources, poor ANC, illiteracy, and a shortage of modern contraceptive practices. The study results indicated a prevalence of grand multiparity (21.09%), which was associated with low education, fewer ANC visits, and adverse maternal and neonatal outcomes [14].

Antenatal care coverage

One of the most important reproductive health indicators is the coverage of ANC, which has been defined as the percentage of women aged 15 to 49 with a live birth in a given period who received antenatal care provided by skilled health personnel at least once during pregnancy. Globally, while 88% of pregnant women access ANC with skilled healthcare personnel at least once, only 66% receive four ANC visits. It is even worse in regions with the highest rates of maternal mortality, such as Western and Central Africa and South Asia, where even fewer women receive at least four ANC visits (53% and 55%, respectively). In Sudan, this indicator was 50.7% during 2012-2014 and 51.2% during 2018-2019 [15]. The most important reproductive health indicators in Sudan were the coverage for the first ANC visit, the coverage for the fourth ANC visit, deliveries in health facilities, deliveries at home, and deliveries with the attendance of skilled health providers. According to unpublished reports from the Federal Ministry of Health, Sudan, Annual Statistical Reports 2021-2022, as shown in Table 1 below, it was obvious that ANC coverage in Sudan was poor even before the current armed conflict.

**Table 1:** The ANC and delivery coverage in Sudan 2021–2022 (MOH-Sudan) ANC: antenatal care; MOH: Ministry of Health

Indicators	2021	2022
Coverage for the first ANC visit	41 %	40%
Coverage of the fourth ANC visit	25%	29%
Deliveries in healthcare facilities	48%	51%
Deliveries at home	52%	49%
Deliveries with attendance of skilled healthcare providers	86%	90%
(Federal Ministry of Health, Sudan. Annual Statistical Reports, 2021- 2022)		

A community-based survey was carried out in 2010 to assess the coverage and identify factors associated with the inadequacy of ANC services in Kassala, eastern Sudan. It revealed low ANC coverage associated with high parity and low education; 90% have only one ANC visit; 11% have four or fewer ANC visits; and 10.0% have not attended at all [16]. Another effect of ANC on teenage primiparous women was studied at Kassala Hospital in Sudan in 2011. The importance of ANC is to provide health education and initiate the concept of contraception, which will help reduce the high incidence of early motherhood and its consequences [17].

Antenatal care providers

Starting from the old and well-established legacy of providing reproductive health services to Sudanese women and passing through the natural development of health services in terms of accuracy and quality, it was expected that today we would celebrate the best and highest quality public health services and reproductive health services in particular, including ANC. Unfortunately, the frequent decline and setbacks took a toll on health services that led to the current deterioration, which reached its end in the war and destroyed what little health services were available.

Antenatal care can be provided by skilled health personnel like doctors, nurses, midwives, traditional birth attendants, medical assistants, community health workers, and health visitors. Traditionally, since the inception of organized health services, ANC has been provided by village midwives, who play an important role in Sudanese women's reproductive health, as evidenced by a 1992 study that assessed the ANC coverage provided by them. The results of this study revealed that 70% of pregnant women contacted the village midwives at least once during their pregnancy; the average attendance was 3.8 antenatal contacts per mother. The village midwives at that time provided the service at their own homes. Fifty percent of these pregnant women were seen in the village midwife's own home, and only 20% were in the mothers' homes. The village midwife attended 76% of the deliveries, 11% were attended by the traditional birth attendants, and the remainder were delivered in hospital. The village midwives demonstrated a reasonable standard of knowledge and competence in various aspects of ANC, and they were socially accepted and appreciated by the community [18]. Another study was conducted in 2014 to evaluate midwifery knowledge of ANC in Omdurman Maternity Hospital, Sudan. Results showed that the majority of the midwives had undergone a two-year midwifery training course and had also had good work experience. They demonstrated good counseling attitudes, but they were deficient in knowledge of the danger signs of pregnancy, nutrition, and infection control. They need close and frequent follow-up and in-service training, with an emphasis on ANC [19]. In 2017, an assessment of community-based ANC services provided by midwives in River Nile State, Sudan, was done. Generally, mothers were satisfied with the ANC provided by midwives; their satisfaction was significantly related to the number of ANC visits and place of examination [20].

Quality of ANC

Quality of healthcare services is one of the most important issues and the main predictor variable to achieve sustainable development goals; it is one of the important steps towards reducing morbidity and mortality [21]. Sudan has one of the highest rates of maternal and perinatal mortality, as we mentioned earlier. One of the success stories in maternal mortality reduction was the health project initiated by the University of Gezira, Sudan, in 2005. The indication was related to village midwives, rural hospitals, the cause of maternal mortality, available obstetrics resources, equipment, manpower, and training in all hospitals in the region. They concluded that effective maternity care capable of reducing maternal mortality and newborn mortality has to be built around ANC with high quality, and the first referral level of care was the 40 rural hospitals. The experiment reported great accomplishments, dropping the maternal mortality ratio (MMR) from 469 per 100,000 live births in 2005 to 139 in 2019, and the neonatal mortality rate (NMR) from 43 per 1,000 live births in 2005 to 12.1 per 1,000 in 2019 [22]. To highlight the problem of ANC quality, in 2022 another study was conducted to assess pregnant women's experience of care and their satisfaction at the primary healthcare center in Gezira, Sudan. They concluded that the poor quality of ANC is due to inadequate material resources, a deficiency of human power, the absence of on-the-job training for service providers, insufficient protocols and guidelines, poor infrastructure, and administrative weakness at health facilities [23]. Another study conducted in 2011 at Ribat University Hospital in Khartoum was conducted to assess the quality of documentation for the ANC card. The obstetrical history documentation was very good; the documentation of the personal history, obstetrical examination, and laboratory investigation were adequate, while the vaccination information was very poor [24].

Accessibility and utilization of ANC

Access to healthcare is the ability to use personal health services in an appropriate way to attain the best possible health outcomes. It is about access to a site of service; entry into the healthcare system requires access to needed services by qualified and trustable providers who meet patients' needs [25]. In other words, it is affluence access in terms of physical access (geographical distribution), costs, time, and availability of qualified personnel. Accessibility is a prerequisite for a high-quality and efficient health system; it can be divided into sub-dimensions such as geographical accessibility, financial accessibility, availability of a qualified workforce, and waiting time [26]. Three studies evaluated access to health services in general and primary healthcare services in particular in Sudan. One was conducted in 2005 among displaced people in Nyala State, South Darfur, to evaluate various health aspects, including women's health, taking into account that among ANC service users 68% did not use contraceptives, and 53% reported at least one birth. Without care, 84% were circumcised, and the prevalence of severe depression was 31%. Women had various unmet health needs and had insufficient rights with regard to marriage, reproductive rights, education, mobility, and access to healthcare, which together may certainly negatively impact health [27]. The other one was a hospital-based study conducted in 2016 among pregnant women attending ANC outpatient clinics at Omdurman Maternity Hospital in Sudan to understand factors influencing the accessibility of pregnant women to ANC services. The majority of the respondents had access to ANC services (93.0%), but they were paid, which may hinder the possibility of using them [28]. The last study was qualitative research about the accessibility and quality of maternal health services in Khartoum in 2018 to investigate why Sudanese women do not attend ANC, their satisfaction, and their views on improving uptake. It demonstrated the factors affecting a woman’s decisions, which may extend beyond physical barriers and include misconceptions, conflict between faith and modern medicine, and dissatisfaction with previously used services. They reported dissatisfaction with care providers’ perceived lack of empathy, unpunctuality, and lack of health promotion, which may also contribute to underutilization [29].

Utilization is the level to which people use services already available in the community, and the appropriate utilization critical to preserving productivity prevents employees from underachieving or being overwhelmed with workloads, leading to burnout. The utilization rate estimates the relationship between a worker's total billable hours and their available hours. The reasons behind the low utilization of ANC services in Sudan have been assessed since 2002; according to Sudanese Ministry of Health reports, it may be due to inaccessibility and a lack of health services, particularly ANC. Hence, there are places where ANC is provided, but attendance is low [30]. During the same year 2002, the routine utilization of ANC services and the application of the tetanus toxoid (TT) vaccination and its associations with socio-economic status, residency, women’s education, quality of care, and walk-time were assessed. Utilization of ANC and TT vaccine applications was higher in urban women as compared to women in rural areas. A higher quality of care, shorter walk-time, and a mother’s educational level were significantly positively associated with more utilization of routine ANC services. Mother’s educational level showed a nearly significant positive relationship with the use of routine ANC services [31]. The determinants of ANC utilization were identified in Marawi, Northern Sudan, in 2015. There was higher utilization (87.4%), but only 46.8% had four or more ANC visits. Distance was the main predictor and was significantly associated with utilization, high cost of transport, waiting time, and other socio-demographic characteristics such as the educational level of women and husbands [32].

Reducing maternal and neonatal morbidity and mortality is a challenge in developing countries. Starting with massive public health interventions to improve ANC utilization will help in solving this challenge by raising the awareness of women of childbearing age about the importance of ANC and providing accessible, high-quality services that induce optimal utilization and maximum benefit for the pregnant woman and her fetus. This will result in a safe delivery, a healthy newborn, and a mother who is aware of and prepared for the postpartum stage. A systematic review and meta-analysis in 2020 examined the effect of ANC utilization on postnatal care service utilization. The pooled estimate of women who had ANC was 1.53 times more likely to have postnatal care; thus, good ANC utilization is one of the strategies for enhancing the utilization of postnatal care services [33]. Another study in Ahfad Family Health Center, Omdurman, Sudan, assessed the factors affecting knowledge and utilization of ANC services in 2023. Although women indicated knowledge concerning the importance of ANC and its relations to the health of the child and mother, economic factors, wait time, and distance of the center from the place of residence (an inhibiting factor) were mentioned as important predictors for the level of utilization [34].

Service components of ANC

The service provided to pregnant women during ANC visits involves screening, treatment of minor illnesses, immunization, dietary monitoring, counseling, and health education. The lifestyle of the pregnant woman must be adapted to the needs of the pregnancy period in terms of physical activity, dietary behaviors, dietary supplements, workload, and rest time.

Antenatal care screening

Antenatal care screening and diagnostic tests can detect pregnancy abnormalities, complications, and fetal anomalies. They are important because they help make sure that the mother and baby are healthy throughout the pregnancy. Different types of tests, including blood tests, urine tests, and ultrasound scans, are used to check different aspects of pregnant women's and their babies' health. While certain tests are administered just to women who are more likely to develop a specific ailment, others are advised for everyone. The ANC tests can help to pick up medical problems early so they can be treated early, and identify any genetic conditions. They check the normal growth and development of the fetus and diagnose some diseases. One of the disorders that often accompanies pregnancy is depression, which has serious consequences for the health of the mother and also has potentially deleterious effects on the developing fetus. In 2012 in Khartoum Maternity Hospital, the prevalence of antenatal depression was 13.4%, ranging from mild to severe. There were significant associations between depression and age, duration of marriage, number of pregnancies, husband's support, and family history of psychiatric disorders [35]. During 2009, the seroprevalence of syphilis was 3% in Khartoum State; most of the investigated were housewives (90.7%); among them, 81.1% had a formal education; 39% knew nothing about syphilis; and only 13.5% knew the right causative agent and information about ways of protection. Fortunately, 63.6% of the respondents knew about the possibility of transmission from the infected pregnant mother to her child. Although syphilis screening was not part of routine pregnancy care screening, this study created evidence of the importance of adding it as a routine screening in accordance with the recommendation of the WHO [36]. Dental health problems accompany pregnancy, and due to hormonal factors (high estrogen and progesterone), women are more vulnerable to periodontal disease. It is an inflammatory disease of the tissues supporting the teeth. In a narrative review in an ANC clinic in Saad Abualila Hospital Khartoum, Sudan, in 2018, results indicated that 24.0% of respondents had periodontal disease, and lower gestational age was associated with periodontal disease, while there was no relationship between periodontal disease and adverse pregnancy outcomes, and periodontal disease treatment during pregnancy does not confer general protection against adverse pregnancy outcomes [37]. Maternal health and nutrition are also important for fetal neurological development, and the high prevalence of iodine deficiency in Sudan is likely to have a significant impact on neurodevelopmental outcomes [38]. Preeclampsia is characterized by hypertension and proteinuria after the 20th week of gestation; it can be diagnosed during pregnancy. apical periodontitis in at least one tooth was found in 54% of the mothers who developed preeclampsia, which is evident that maternal apical periodontitis may be a strong independent predictor of preeclampsia [39]. Proper ANC can help in managing and reducing the risk of dental health problems through comprehensive dental examinations for detecting and treating them and an effective referral system to dental care services before and during pregnancy.

Dietary monitoring

In the Bahry Locality of Khartoum Sudan in 2010, more than half of pregnant women were anemic, with high-risk pregnancies mostly affected by maternal education, occupation, husband’s education, poor spacing between pregnancies, and the dietary pattern of intake of foods rich in iron. On the other hand, they still had insufficient coverage and inadequate utilization of ANC services [40]. The WHO recommends that all pregnant women in areas where anemia is predominant receive supplements of iron and folic acid, which are protective against maternal anemia and low birth weight (LBW) infants. In 2014 in Khartoum, Sudan, 92.1% of pregnant women used iron-folic acid supplementation during pregnancy, and 65.4% used folic acid. Utilization of ANC, primiparity, maternal employment, and old age were associated with iron-folic acid use [41]. Receiving maternal supplementation such as docosahexaenoic acid, folic acid, vitamin D, and iodine within recommended safe intakes with close medical supervision in populations with dietary deficiencies may prevent many brain and central nervous system malfunctions and even enhance brain development and function in their offspring [42]. In 2009, at Madani Hospital, Sudan, 12.6% of the neonates were LBW. The main risk factors were lack of ANC and maternal anemia, while maternal socio-demographic characteristics (age, parity, and mother education) and anthropometric measurements were not associated with LBW [43].

Vaccination

Tetanus toxoid vaccination during pregnancy is an indispensable component of ANC and has been proven as a safe and cost-effective preventative measure against tetanus deaths and to reduce the incidence of maternal and neonatal morbidity and mortality worldwide [44]. The WHO initiated the maternal and neonatal tetanus elimination initiative to reduce cases until it is not a main public health problem (less than one case of neonatal tetanus per 10,00 live births in every district). Although TT vaccination is provided for pregnant women as well as children as part of a nationwide vaccination policy, Sudan is one of 39 countries that have yet to eliminate maternal and neonatal tetanus [45]. The Simple Spatial Survey (S3M) reported in 2020 that only 67.34% of the women received two doses of the TT vaccine during their last pregnancy [46]. In 2020, in Khartoum, only 40% of the pregnant women had received three doses or more of the TT vaccine during ANC visits. The study reveals that a lower-than-expected number of pregnant women received the TT vaccine, which raises the risk of neonatal tetanus in unborn children. In addition, mothers who have five children or more have a 10-fold greater opportunity to be vaccinated [47].

Adequate tetanus vaccination among women of childbearing age means receiving two doses of the TT vaccine in the last pregnancy, more than one dose within the last three years of birth, more than two doses within the last five years of birth, more than three doses within the last 10 years of birth, or more than four doses at any time. In Sudan, in 2014, the prevalence of mothers who had adequate tetanus vaccination was 60.0%. Antenatal care tetanus vaccination was significantly associated with having four or more ANC visits, a higher level of mothers’ education, a higher household wealth index, and living in areas with a low intensity of armed conflicts. Socioeconomic status had a significant impact on adequate ANC tetanus vaccination, which shows that there is unequal access to tetanus vaccination among women of childbearing age in Sudan [48]. In the Omdurman Maternity ANC clinic in Sudan in 2013, levels of ant-tetanus immunity, adherence to vaccination schedules, and the application of booster doses when needed were assessed. The rate of full protection was 69.2%, and the concentration of ant-tetanus antibodies was significantly associated with the vaccination status, number of TT doses, and duration of the elapsed time since the last TT dose increased the susceptibility of tetanus [49].

Counseling and health education

The level of pregnant women's knowledge about the required dietary intake during pregnancy and even their awareness of expected danger signs has a great influence on a safe and positive pregnancy experience; all of this can be acquired during ANC visits. In Kassala, eastern Sudan, during 2010, only 16.2% had ≥ four ANC visits, while 83.8% had at least one visit. The duration of the ANC visit did not exceed five minutes or less; only 10.6% recalled that they were educated about diet and nutrition, 28.4% on pregnancy symptoms, 45.4% on the schedule and timing of the subsequent visit, 63.4% on the plan of delivery, and 4.1% on family planning. However, no woman had been counseled about screening for sexually transmitted infections (STIs), HIV, or breast self-examination. Most of the women (88.1%) were not aware of the danger signs of pregnancy [50].

Antenatal care during political instability and conflicts

The health situation in Sudan has been seriously affected by the ongoing war. The WHO report on June 20, 2023, indicated that about 66% of Sudanese hospitals in areas affected by the ongoing fighting are out of service, and some maternity hospitals are closed. Eleven million Sudanese people need essential health aid; 2.64 million of them are women and girls of reproductive age; about 262,880 of them are pregnant; and over 90,000 will give birth in the next three months. [51]. The previously mentioned report by the WHO in June 2023 about women and girls in Sudan reported the critical current situation. Numerous studies and reports since 2013 have systematically documented political instability, armed conflict, and their impact on the health system. In a study conducted in 2013 to comprehend the effects on health of both inequality and political armed conflict in Sudan, data from SHHS2 2010 was used to evaluate the role of both asset distribution and armed conflict in health-related outcomes. The results about food consumption, life expectancy, teen births, and infant mortality showed that outcomes were significantly worse in the states with more skewness in wealth distribution. Researchers concluded that wealth inequality and armed conflict are associated with poor population health in Sudan. To address resource distribution and related health issues, policies, and public health strategies are required. Wealth redistribution in the more unequal states, as well as a political resolution of conflict, may improve population health [52].

The military coup in Sudan in October 2021 was one of the periods of political instability and had obvious consequences for the national health system in general and reproductive health in particular. The weakness of the health system has certainly led to the extension of health disparities among the Sudanese people, especially the vulnerable groups [53]. The preterm birth rate in Sudan is estimated to be 13.3%. Accurately defining perinatal outcomes and relating them both to events in the pregnancy and the eventual infant outcomes is a critical step in the development of interventions to improve perinatal and population health. As the burden of disease in Sudan is always affected by poverty and is complicated by geography, politics, armed conflict, and mismanagement, it remains high during political transition time [13].

A series of papers by Bendavid et al., published in 2021, focused on the direct and indirect health effects of armed conflict on women and children. They mentioned several serious facts in this regard. Women and children bear considerable morbidity and mortality as a result of armed conflicts. According to international databases of refugees and internally displaced populations worldwide, nearly 36 million children and 16 million women were estimated to be displaced in 2017. From 185 million women and 250 million children in 2000, the number of non-displaced women and children living dangerously close to armed conflict (within 50 km) increased to 265 million women and 368 million children in 2017. Women’s and children’s mortality risk increases considerably in response to nearby conflict. Between 1995 and 2015, there were over 10 million deaths worldwide among children under the age of five, which can be linked to conflicts. Women of reproductive age living near high-intensity conflicts have three times higher mortality rates than women in peaceful settings. The number of women and children affected by armed conflict has grown steadily since 2000, due to different reasons. How health can be affected by conflict is variable, but systematic evidence links conflict to malnutrition, physical injuries, acute and infectious diseases, poor mental health, and poor sexual and reproductive health. Clearer information on the indirect health effects of armed conflicts could greatly help in the design and implementation of vital interventions for reducing the harms of armed conflicts. Women and children who experience conflict and displacement are more susceptible to harassment, early marriage, sexual assault, isolation, and exploitation. Several studies mentioned that maternal mortality is increased by 28% in relatively more intense conflicts compared with conflict-free periods and that fertility in conflict settings might decrease because of demographic changes such as reduced frequency of marriages and spousal separation and biological effects like reduced fecundity or increased abortion. Contrariwise, reduced access to modern contraceptives coupled with increased sexual violence might increase the number of unintended pregnancies and abortions [54]. The current war in Sudan has had a disastrous impact on maternal and perinatal health. When pregnant women are exposed to armed conflicts, they will face unsafe delivery and many adverse pregnancy outcomes, including miscarriage, stillbirth, prematurity, and low birth weight. Regrettably, that is the fate of pregnant Sudanese women during this current war; moreover, war-related sexual and gender-based violence has been reported [55].

The destruction of healthcare facilities and infrastructures during the war will lead to ineffective, inaccessible, and unutilized health systems and a deficiency of skilled health workers, and the undoubted consequences will be unfortunate maternal and perinatal outcomes [56]. Sudan has a long history of armed conflicts associated with poor health situations. A multiple-indicator cluster survey was used in 2014 to explore the level of ANC tetanus vaccination in the country and to identify the influencing factors such as residency in the armed conflict intensity areas. The study created this parameter by using the scale of the Heidelberg Institute for International Conflict Research (HIIK), which classified the armed conflict intensity in South Kordofan, Blue Nile, and the five states of Darfur as high. Regarding the area of residence, the results showed no significant difference among the participants from rural and urban areas. Nevertheless, compared to other groups, women from locations with lower severity of violent conflicts had significantly greater rates of antenatal tetanus vaccination. However, the armed conflicts in the case of Sudan pose further challenges since these high-risk areas are often geographically remote with poor infrastructure and health systems. Inequalities in healthcare could threaten the overall growth and development of the country [48]. Sexual violence is very often used as a weapon of war against young girls to humiliate an ethnic group, intimidate them, or spread terror among civilians. These actions usually result in long-lasting consequences, such as psychological scars or pathological conditions, and even have social and economic detrimental effects. Young girls, due to their young, not-fully-developed bodies, might be affected by vaginal fistulae, uterine prolapse, and other complications, in addition to being exposed to sexually transmitted diseases, early pregnancies, and unsafe abortions [57]. The flared-up armed conflict in Sudan in April 2023 resulted in many victims, internal and international displacement, and refugees. Sudan’s health system was severely affected by frequent attacks on healthcare facilities and workers; many hospitals have been closed, and numerous medical facilities have been occupied by armed groups. Vital services have been suspended, disrupting health services in conflict areas and increasing strain on neighboring regions’ healthcare facilities. The conflict also caused shortages of necessary medical supplies, plunder of healthcare facilities and humanitarian supplies, and destruction of infrastructure, affecting the supply chain and availability of healthcare resources. The financial challenges are expected to intensify. The conflict has also worsened the situation of diseases, with possible outbreaks of dengue fever, measles, and spikes of gender-based violence reported. The restoration and maintenance of the healthcare system are crucial through the coordinated efforts of the Sudanese Ministry of Health and international partners [58].

## Conclusions

According to the reviewed data, ANC services in Sudan, even during normal political situations, were not sufficient and were of poor quality in most of the reviewed regions. The political instability and armed conflicts worsened the situation, and it became catastrophic. We recommend some steps to strengthen the available services and to provide minimally required services during emergencies or wartime. They are as follows: improving the accessibility and quality of ANC services by governmental health authorities and all stakeholders; focusing on addressing the healthcare needs of vulnerable groups, remote, rural areas, and nomadic communities in providing the needed healthcare services in general and ANC in particular through outreach services; fortifying and updating the healthcare providers' skills through continuous on-the-job training and close monitoring; revising and updating the working procedures guidelines to match the needs of the local communities; raising community awareness about the importance of ANC and its effect on mothers' and newborns' health; and providing effective, punctual, and applicable contingency plans for abnormal situations to replace routine services during crises. More research is needed to cover the gaps and to help in developing evidence-based plans for the future.
